# Protocol to study recurrence of *Clostridioides difficile* infection in murine model with built-in colonization and contamination controls

**DOI:** 10.1016/j.xpro.2026.104746

**Published:** 2026-08-22

**Authors:** Marjorie Pizarro-Guajardo, Trey Hejtmancik, Daniel Paredes-Sabja

**Affiliations:** 1 Department of Arts & Sciences, Texas A&M University, College Station, TX 77843, USA

**Keywords:** Immunology, Microbiology, Model Organisms

## Abstract

Pre-colonization with endogenous murine *Clostridioides difficile* strains and cross-contamination between animals can compromise recurrent *C. difficile* infection (R-CDI) studies. We present a murine R-CDI protocol with built-in colonization and contamination controls. Mice receive an antibiotic cocktail and clindamycin before challenge with *C. difficile* spores, followed by vancomycin to induce recurrence. Selective cycloserine-cefoxitin-fructose agar (CCFA)/taurocholate-CCFA (TCCFA) fecal cultures quantify colonization, while PCR ribotyping identifies endogenous contaminants as a quality control. A two-handler workflow minimizes cross-contamination.

## Highlights

• Steps for a standardized murine model of recurrent *C. difficile* infection
• Detection of endogenous *C. difficile* pre-colonization in key steps
• Two-handler workflow minimizes cross-contamination between animals
• Instructions for ribotyping colonies to confirm infecting strain and exclude contaminants

## Abstract (editor highlights)

Publisher’s note: Undertaking any experimental protocol requires adherence to local institutional guidelines for laboratory safety and ethics.

## Graphical abstract

**Figure:**
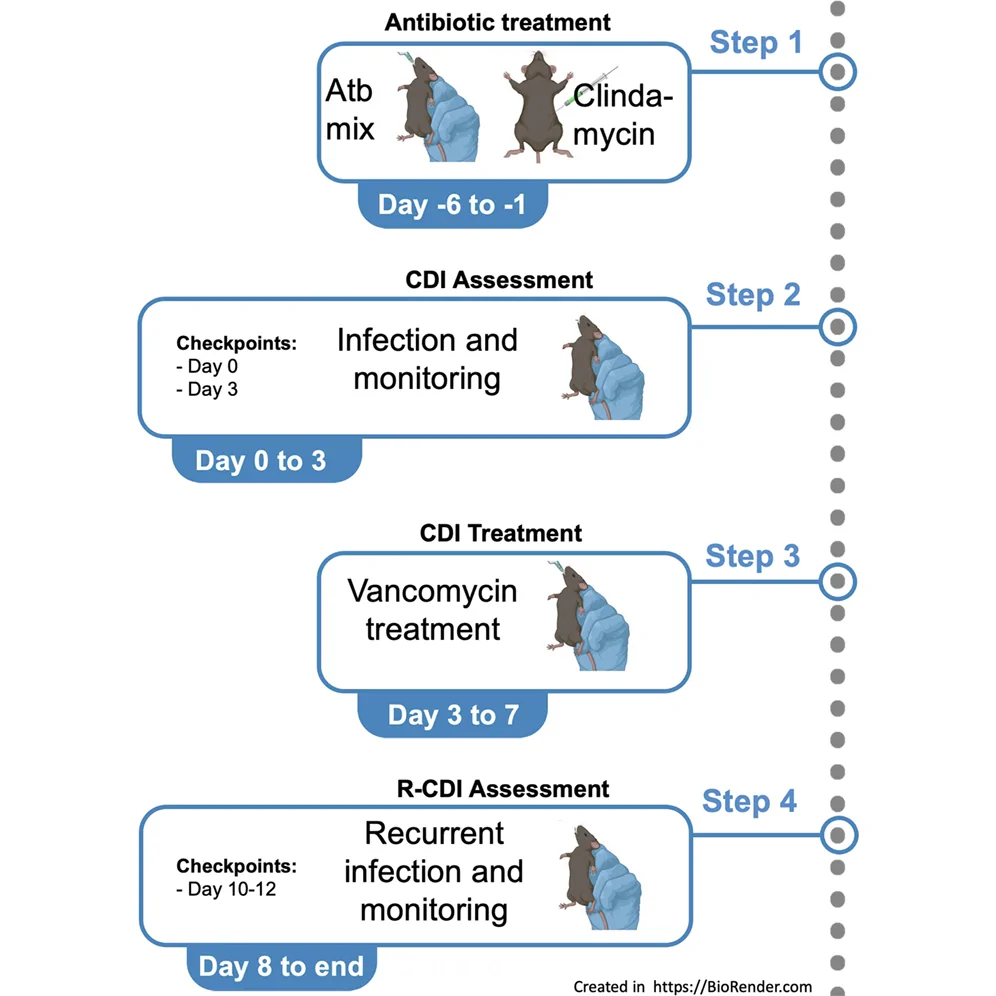

## Before you begin

### Innovation

Study of recurrent *C. difficile* infection (R-CDI) in mice remains a major challenge due to pre-colonization of mice with endogenous, most likely non-toxigenic, *C. difficile* strains, which can prevent the manifestation of CDI symptoms after experimental challenge.^1^ Endogenous *C. difficile* strains have been reported in 20% to 50% of animals, typically detected as weight loss after antibiotic treatment and a bloom in colonization upon antibiotic exposure,^2^^,^^3^ causing animal to shed spores, contaminate cage-mates, and alter infection outcomes.^1^

Our protocol directly addresses this experimental challenge by incorporating standardized pre-challenge screening for endogenous *C. difficile*, coupled with built-in microbiological checkpoints at key stages of the experiment (baseline, post-antibiotic treatment, primary infection, and recurrence) to detect silent colonization and cage-level cross-contamination. Step-by-step guides for rigorous handling procedures, including dedicated cage-changing workflows, segregation of treatment groups, and routine culture or qPCR of fecal sentinel samples, are provided to minimize horizontal transmission, thereby reducing noise in colonization and disease-readout data and providing a reproducible, quality-controlled R-CDI experimental platform.

### Institutional permissions

All animal husbandry and experiments were authorized by the Texas A&M University Institutional Animal Care and Use Committee (IACUC) 2024-0154.

### Acquisition of institutional approval

**Timing: Up to several weeks**

1. Obtain approval from the institutional animal care and use committee (IACUC) or the equivalent regional authority for all procedures involving mice and infection with *C. difficile*, a biosafety level 2 (BSL2) agent.

***Note:*** The protocol should cover antibiotic treatment, *C. difficile* challenge, monitoring of clinical signs, and humane endpoints (maximum allowed weight loss and clinical score thresholds).

### Set up animal housing and biosafety practices

**Timing: 1–2 days (excluding acclimatization)**

2. House C57BL/6J mice individually in ventilated cages under specific pathogen–free conditions, with controlled temperature, humidity, and a 12 h light/dark cycle. Allow animals to acclimatize for at least 7 days before initiating antibiotic treatment.
3. Perform all procedures involving *C. difficile* or contaminated materials in a certified Class II biosafety cabinet using appropriate personal protective equipment (gown, gloves, mask, and eye protection). Establish a unidirectional workflow to separate “clean” and “contaminated” areas.

### Train and organize personnel

**Timing: 1–2 weeks (training period)**

4. Ensure that all personnel listed in the protocol have completed the required training in animal handling, BSL2 practices, and euthanasia methods, and that approval numbers and expiration dates are recorded in the laboratory documentation.
5. Train at least two people per experimental session in key techniques: oral gavage, intraperitoneal injection, collection of fecal pellets by gentle abdominal massage, and euthanasia of moribund animals. Practice on non-infected mice until procedures can be performed reliably and with minimal distress.
6. Define roles for each person (e.g., **animal handler** and **clean handler**) to maintain efficiency and reduce handling time per cage, and instruct all personnel on clinical scoring, weight monitoring, and criteria for euthanasia.

### Prepare disinfectant solution

**Timing: 10–15 min**

7. Prepare a 10% (v/v) bleach solution from commercial stock in clearly labeled bottles.^4^
  ***Note:*** Dilute 1 part household bleach to 9 parts water.
  a. Annotate preparation date and expiration date 7 days later, as diluted bleach loses activity over time.
  b. Maintain a minimum contact time of 5 minutes for all contaminated surfaces.
    **Alternatives**: Perisept can be used as alternative surface disinfectant. It is corrosive and can cause irreversible eye damage, skin burns, damage if inhaled or swallowed. Apply only with appropriate protection and in well-ventilated environments, ensuring that animals are housed in a separate room.

### *C. difficile* spore preparation

**Timing: 9 days**

8. Generate and store *C. difficile* spores using a validated sporulation protocol under biosafety level 2 (BSL2) conditions. Prepare working aliquots to avoid repeated freeze-thaw cycles.^5^
  ***Note:*** We recommend using the protocol described by Weldy, et al.^5^ or the steps detailed below.
  a. Culture *C. difficile* R20291 in Brain Heart Infusion (BHIS) broth for 16 h in anaerobic chamber at 37°C to obtain a stationary-phase culture.
  b. Inoculate 250 μL stationary-phase culture onto 70:30 agar plates and spread with L-shaped cell spreader.
  c. Incubate plates at 37°C for 5 days in anaerobic conditions.
  d. Scrape plates with inoculating loops and collect bacterial material into 1.5 mL tubes with sterile water.
  e. Centrifuge the tubes, and discard the supernatant. Resuspend in 1 mL PBS and store at 4°C for 16 h under aerobic conditions to allow cell lysis.
  f. Centrifuge the tubes at 18,407 × *g* for 5 min, discard the supernatant and resuspend in 1 mL of PBS. Repeat this washing step 10 times, until 99% purity is obtained.
    ***Note:*** After several washes, the pellet will appear fractionated, and upper white layer can be carefully removed.
  g. Count spores and adjust to 1 × 10^9^ spore/mL. Store at −80°C in aliquots of 50 μL.
9. Before initiating the mouse experiment, determine the spore viability of one of the spore aliquots, by thawing one tube and plating serail dilutions on 0.1% Taurocholate-BHIS (T-BHIS) agar plates. Adjust the stock concentration to CFU/mL.

***Note:*** Spore viability is affected by long term storage at −80°C and repeated freeze-thaw cycles. Spores stored at −80°C can be used up to 2 months, and spore viability must be quantified 1 week before infection.

### Pre-weigh collection tubes

**Timing: 30–45 min**

10. Record the weight of sterile 1.5 mL tubes using an analytical balance. Prepare one tube per mouse per day, plus an additional 20% to account for losses. Record the tare weight directly on the tube lid.

### Prepare selective agar plates for *C. difficile* vegetative cell and spore enumeration

**Timing: 3 h**

11. Prepare *C. difficile*–selective cycloserine-cefoxitin-fructose agar (CCFA) and taurocholate-CCFA (TCCFA).
  a. Prepare 72 g of proteose peptone in 1 L of water.
  b. Adjust the volume to 1,4 L, transfer to a 2 L Erlenmeyer flask, and add 21.6 g of agar.
  c. Autoclave for 40 min and cool to 55°C.
  d. Add supplements as described in material and methods (solutions).
  e. Pour approximately 20–25 mL per 90 mm plate under sterile conditions.
12. Allow plates to solidify and dry with lids slightly ajar in a biosafety cabinet until the surface is free of visible moisture (20 min). Store plates at 4°C in sealed bags for up to 2 weeks, and warm them to 24°C inside the biosafety cabinet before use.

### Synthetize primers for ribotyping PCR

**Timing: Depends on shipping time**

13. To distinguish pre-colonizing strains from the R20291 strain used for infection, PCR ribotyping primers will be used. These primers amplify the variable-length intergenic region between 16S and 23S rRNA genes. Forward 16S rRNA “GTGCGGCTGGATCACCTCCT”, Reverse 23S rRNA “CCCTGCACCCTTAATAACTTGACC”.^6^

## Key resources table

**Table:** 

REAGENT or RESOURCE	SOURCE	IDENTIFIER
**Bacterial and virus strains**		

*C. difficile*	ATCC	BAA-1805

**Chemicals, peptides, and recombinant proteins**		

Bacto Proteose Peptone	BD	211684
Difco Agar Technical	BD	281230
Na_2_HPO_4_ dihydrate	Supelco	7588-79-4
Brain heart infusion	BD	90000-060
Yeast extract	Gibco	DF0127179
KH_2_PO_5_	Millipore	7778-77-0
NaCl	Thermo Scientific	A17718.0I
MgSO_4_	Millipore	10034-99-9
KCl	Sigma	P3911-1KG
Fructose	Thermo Scientific	AAA177180I
Sodium taurocholate	Sigma	86339-25G
L-cysteine	Sigma	C7880-100G
Taq DNA Polymerase	New England Biolabs	M0267
Sodium chloride 0.9%	VWR	102979-272
Kanamycin sulfate	Sigma	90915-25G
Gentamycin sulfate	Thermo Scientific	455310050
Colistin sulfate salt	Sigma	C4461-1G
Metronidazole	Sigma	M1547-25G
Clindamycin hydrochloride monohydrate	Thermo Scientific	J61409.03
Vancomycin	Thermo Scientific	J62790.06
Cycloserine	CHEM-IMPEX	00618
Cefoxitin	CHEM-IMPEX	01490

**Other**		

Biosafety cabinet BSL2 A2	Labcono	302619101
Thermal cycler	Analytik Jena	Biometra Trio 30
Water bath	VWR	VBE20
Anaerobic chamber	Coy	Type B
Analytical balance	VWR	164AC
Ultrapure Water Purification System	MiliQ	IQ7000
Gavage needles	Burgoon Company EVCO Partners LP DBA	5FTV4
1cc syringe (27G × ½)	BD	309623
0.22 um syringe filter, Nylon	VWR	76479-030
L-shape cell spreader	VWR	76207-748
Inoculating loops	VWR	12000-810
2L Erlenmeyer flask	N/A	N/A
Petri dishes	VWR	25384-088
1.5 mL tubes	VWR	20170-038
96-well plate	Falcon	087722C
Bleach	Clorox	N/A
Ethanol analytic grade	Fischer	A405F-1GAL

**Oligonucleotides**		

Forward 16S rRNA: GTGCGGCTGGATCACCTCCT	Ref. 6	N/A
Reverse 23S rRNA: CCCTGCACCCTTAATAACTTGACC	Ref. 6	N/A

## Materials and equipment

### Solutions

***Note:*** Prepare all solutions and media with type 1 ultra-pure water (resistivity of 18 MΩ × cm), as salts present in tap or distilled water can impair *C. difficile* growth.

***Note:*** Prepare all antibiotic solutions fresh before administration and store at 4°C until use to preserve antimicrobial activity.

**Table:** Antibiotic mix

Reagent	Final concentration in the mix
Kanamycin sulfate (40 mg/kg)	8 mg/mL
Gentamycin sulfate (3.5 mg/kg)	0.7 mg/mL
Colistin sulfate salt (4.2 mg/kg)	0.84 mg/mL
Metronidazole (21.5 mg/kg)	4.3 mg/mL
Vancomycin (4.5 mg/kg)	0.9 mg/mL

Calculate the total volume needed each day to dose all mice, plus the equivalent volume for 2 additional mice (250 μL per mouse per administration to account for dosing losses). For example, 4 mice require 1.0 mL; therefore, prepare 1.5 mL. Dissolve the required amounts of each antibiotic in sterile 0.9% NaCl, mix gently until fully dissolved, and filter-sterilize through a 0.22 μm filter.

• Clindamycin solution.

Calculate the total volume needed for the entire course (250 μL per mouse per administration to account for dosing losses). Prepare 2.0 mg/mL clindamycin working solution to achieve the desired dose of 10 mg/kg. Dissolve clindamycin in sterile 0.9% NaCl and filter-sterilize through a 0.22 μm filter.

• Vancomycin solution.

Prepare 10.0 mg/mL vancomycin working solution to achieve the desired dose of 50 mg/kg in a final volume of 250 μL per mouse. Dissolve vancomycin in sterile 0.9% NaCl thoroughly and filter sterilize through a 0.22 μm filter.

**Table:** PBS

Reagent	1 L 10× PBS	Final concentration
NaCl	80 g	1.37 M
KCl	2 g	27 mM
Na_2_HPO_4_	14.4 g	100 mM
KH_2_PO_4_	2.4 g	18 mM
pH 7.4	–	–

To make 1 L of 10× PBS, dissolve all the components in approximately 800 mL of water, adjust the pH to 7.4 and add water to a final volume of 1 L.

For 1× PBS, dilute 100 mL of 10× PBS with 900 mL of water.

Store at 24°C for 12 months.

• CCFA and TCCFA plates.

***Note:*** This recipe is designed to generate 1.8 L of agar, which yields approximately 90 plates.

***Note:*** One CCFA and one TCCFA plate is required per mouse per day.

**Table:** 5× salts

Reagent	1L 5× salts	Final concentration
Na_2_HPO_4_	25 g	140 mM
NaCl	10 g	171 mM
KH_2_PO_4_	5 g	37 mM
MgSO_4_	0.5 g	4.2 mM
pH 7.4	–	–

Dissolve the components in 1 L of water to prepare the 5× salts solutions. Filter-sterilize. This solution can be stored at 24°C for 6 months.

• 30% fructose.

Dilute 10.8 g of fructose in 36 mL of water to prepare a 30% fructose solution. Filter-sterilize. This solution can be stored at 24°C for 3 months.

**Table:** Proteose peptone agar

Reagent	1.4 L	Final concentration
Proteose peptone	72 g	5.14% w/v
Agar	21.6 g	1.54% w/v

Dissolve 72 g of proteose peptone in 1 L of water, then adjust to 1.4 L, transfer to a 2 L Erlenmeyer flask, and add 21.6 g of agar. Autoclave for 40 min and keep in a 50°C water bath until use. Do not allow the medium solidify, use it on the same day.

• Cycloserine.

Dilute 1 g of cycloserine in 10 mL of water to make a 100 mg/mL cycloserine solution. Filter-sterilize. This solution can be stored at 4°C for up to 2 days or at −20°C for up to 1 month.

• Cefoxitin.

Dilute 160 mg of cefoxitin in 10 mL of water to make a 16 mg/mL cefoxitin solution. Filter-sterilize. This solution can be stored at 4°C for up to 2 days or −20°C for up to 1 month.

**Table:** CCFA and TCCFA Plates

	Final concentration	Volume to 1.8 L	Volume to 1.0 L
10% Sodium taurocholate^a^	0.1%	18 mL	10 mL
100 mg/mL cycloserine	250 μg/mL	4.5 mL	2.5 mL
16 mg/mL cefoxitin	16 μg/mL	1.8 mL	1.0 mL
5× salts	1× salts	360 mL	200 mL
30% Fructose	0.6% Fructose	36 mL	20 mL
Proteose peptone-agar	40 g/mL	1400 mL	777.8 mL

Mix all components at 50°C and pour 20–25 mL per plate. This formulation yields 1.8 L which produces approximately 90 plates. One CCFA and one TCCFA plate are required per mouse per day. Store plates at 4°C for 2 weeks.

a Add sodium taurocholate only to TCCFA plates to induce spore germination.

## Step-by-step method details

### Dynamics of *C. difficile* CDI and R-CDI

**Timing: 21 days**

Day-by-day activities in the *C. difficile* infection in mice model to study R-CDI.

***Note:*** On days −6 to −4, administer the antibiotic mix by oral gavage. After 2 days of observation, on day −1, administer clindamycin by intraperitoneal injection. On day 0, challenge with *C. difficile* spores. Between days 3 and 7, administer vancomycin by oral gavage (Table 1; Tables S1 and S2). Monitor clinical scores every day of the experiment (Table 4). Monitor colonization from day 0 to the end of the experiment. Evaluate ribotype of grown colonies from days 0, 3 and 11.

**CRITICAL:** Two individuals are needed to handle mice. The animal handler is exposed to contamination by manipulation of animals inside a Class II biosafety cabinet and must avoid touching any external cage surfaces or equipment that could lead to environmental contamination outside the biosafety cabinet. The clean handler moves cages in and out the cabinet, decontaminates surfaces when needed, prepares dosing needles, records clinical scores (Table 2). To prevent cross-contamination, the animal handler must change gloves after handling each mouse.

***Note:*** To test for endogenous *C. difficile*, at each timepoint, culture fecal sample on CCFA and TCCFA media and perform ribotyping.

***Note:*** To monitor cross-contamination, sentinel animals (treated with antibiotic and not infected) can be placed alternated among infected animal cages. Work on one animal at the time, introducing one cage in the biosafety cabinet at the time.

1. Antibiotic mix (days −6 to −4, Table 3).
  a. Weigh each mouse and collect clinical score (Table 4).
  b. Administer the appropriate volume of the antibiotic mix via oral gavage, calculated according to the animal’s body weight (Table 5).

**Table 5:** Dosage volume according to animal’s body weight

Mouse weight	Volume of gavage
22 g	110 μL
20 g	100 μL
18 g	90 μL
16 g	80 μL

***Note:*** Mice can be caged in groups throughout antibiotic mix administration and recovery days (step 2).

**CRITICAL:** The assessment of R-CDI incidence and persistence requires that mice are individually caged after infection day, due to risk of re-infection by coprophagy.

2. Recovery (days −3 to −2, Table 3).
  a. Weigh each mouse and collect clinical score (Table 4).
3. Intraperitoneal injection of clindamycin (day −1).
  a. Weigh each mouse and collect clinical score (Table 4).
  b. Administer the appropriate volume of clindamycin via intraperitoneal injection, calculated according to the animal’s body weight (Table 5).
  c. Change cages.
  d. Place PBS and pure ethanol in the anaerobic chamber for use throughout the experiment, and CCFA/TCCFA plates (one plate of each per mouse) for use the next day.

***Note:*** PBS and ethanol must stay in the anaerobic chamber for at least 16 h before use to allow reduction of the solvents and prevent damage to vegetative cells contained in the feces.

4. Spore challenge via oral gavage (day 0, Table 3).
  a. Weigh each mouse and collect clinical score (Table 4).
  b. Collect a fresh stool from the animal in regular sterile 1.5 mL tube. Bleach the surface of the tube.
  c. Administer the 100 ul of spore suspension via oral gavage, calculated according to the animal’s body weight.
  d. Place CCFA/TCCFA plates in the anaerobic chamber (one plate of each per mouse) to next day.
  e. Evaluate *C. difficile* pre-colonization in anaerobic chamber (described below).

***Note:*** In our experience, endogenous *C. difficile* is detectable mostly after clindamycin. The day of infection is the first trustable checkpoint, as in previous days, mice do not shed detectable levels of these spores.

**CRITICAL:** Fresh stools must be collected before the infection to prevent contamination and false positives. Culture stools in CCFA and TCCFA the day of collection to prevent sample accumulation and prevent viability loss of vegetative cells due to oxygen. Vegetative cells within the stools can stay in aerobic atmosphere for a maximum of 1 h before they lose viability in the counts.

***Note:*** On day 0, pre-contamination needs to be assessed following the quantification of colonization (steps 8–14), avoiding the serial dilution (skipping steps 10.c and 11.c).

***Note:*** If a more severe or less severe infection is desired, the dose infection must be evaluated aiming to obtain 100% onset of infection and 100% survival at the last day of vancomycin to allow observation of R-CDI development. This is relevant when mutant strains or different ribotypes are tested.

**Figure 1:**
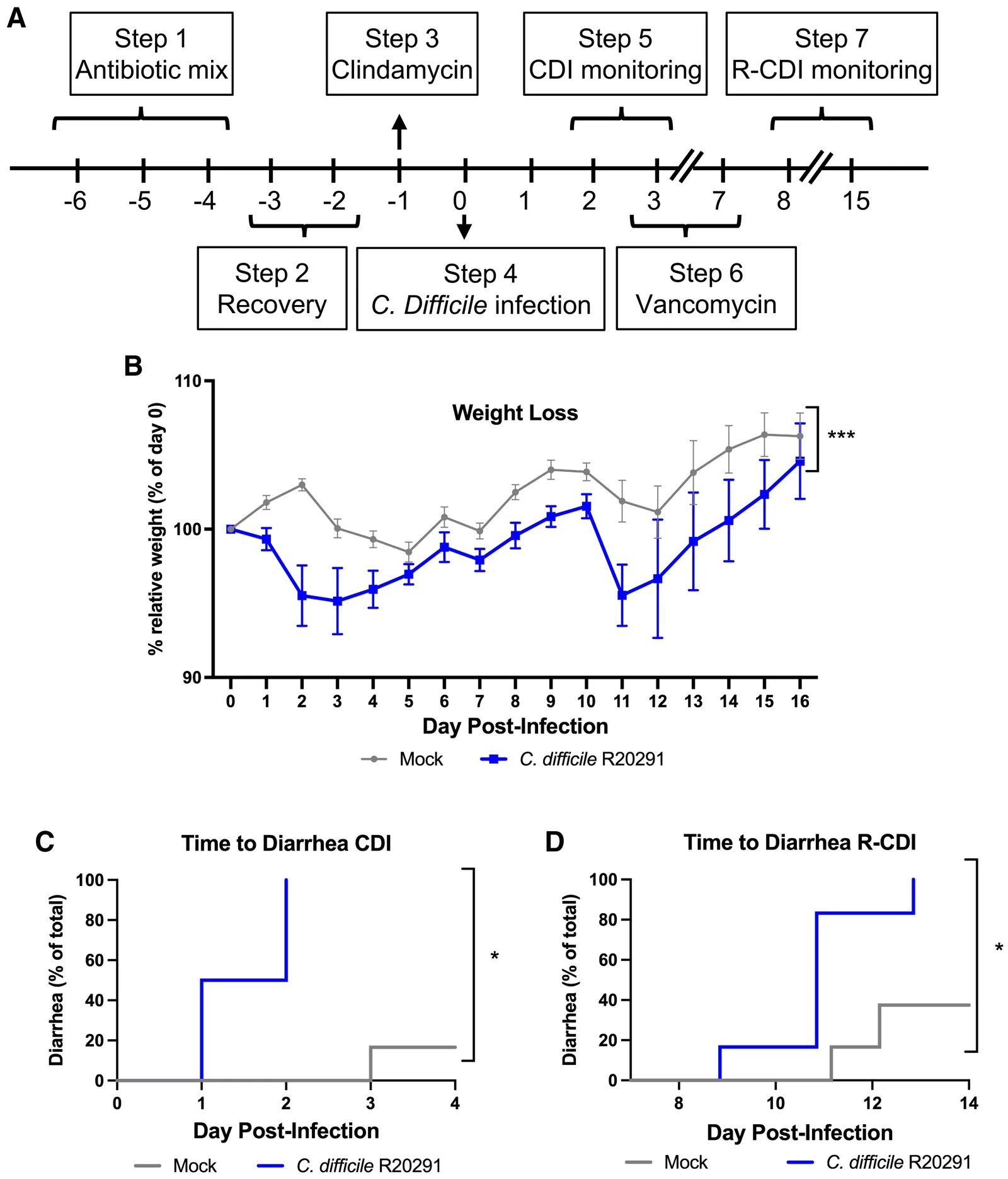
Assessment of *C. difficile* R-CDI infection after filtering out pre-contaminated animals (A) Timeline of activities to determine dynamics of *C. difficile* Infection and induction of R-CDI. Mann-Whitney test, ∗∗∗ = *p* < 0.001. Error bar represents SEM. (B) Relative weight loss (average + SEM), (C) time to diarrhea in CDI and (D) R-CDI. N = 6. Log-rank (Mantel-Cox) test, ∗ = *p* < 0.05.

5. CDI monitoring (days 1 to 2, Table 3).
  a. Weigh each mouse and collect clinical score (Table 4).
  b. Collect a fresh stool from the animal in pre-weighted sterile 1.5 mL tube. Bleach the surface of the tube.
  c. Place CCFA/TCCFA plates in the anaerobic chamber (one plate of each per mouse) for use the next day.
6. Remission induction by vancomycin treatment (day 3 to day 7, Table 3).
  a. Weigh each mouse and collect clinical score (Table 4).
  b. Administer the appropriate volume of vancomycin by oral gavage, calculated according to the animal’s body weight (Table 5).
  c. Collect a fresh stool from the animal in pre-weighted sterile 1.5 mL tube. Bleach the surface of the tube.
  d. Change cages.
  e. Place CCFA/TCCFA plates in the anaerobic chamber (one plate of each per mouse) to next day.
  f. Quantify colonization in anaerobic chamber (described below).
7. R-CDI monitoring (day 8 to 15, Table 3).
  a. Weigh each mouse and collect clinical score (Table 4).
  b. Collect a fresh stool from the animal in pre-weighted sterile 1.5 mL tube. Bleach the surface of the tube.
  c. In the anaerobic chamber, place PBS, pure ethanol and CCFA/TCCFA plates (one plate of each per mouse) for use the next day.
  d. Quantify colonization in anaerobic chamber (described below).
8. Graph weight loss normalized to Day 0 weight and time to diarrhea (Figure 1).

**Table 1:** Experimental overview and timeline of CDI and R-CDI

Day	Treatment, route	Timepoints for colonization	Timepoints for ribotyping
−6 to −4	Antibiotic mix, gavage	N/A	N/A
−2, −3	Observation	N/A	N/A
−1	Clindamycin, i.p.	N/A	N/A
0	Challenge with 10^6^*C. difficile* spores, gavage	Only spread plate	N/A
1, 2	Observation	Spread plate and serial dilutions	N/A
3–5	Vancomycin, gavage	Spread plate and serial dilutions	D3: maximum symptoms CDI
8–12	Observation	Spread plate and serial dilutions	D11: maximun symptoms R-CDI

**Table 2:** Roles of clean and contaminated handlers

Operation order	Staff	Description activities
1	Clean Handler	Bring the cage into the BSC and open the lid.
2	Animal Handler	Assess symptoms and behavior score.
3	Animal Handler	Measure animal weight.
4	Animal Handler	Assess diarrhea score and collect stool.
5	Clean Handler	Record scores and weight.
6	Clean Handler	Prepare dosing syringe (antibiotic mix, infection and vancomycin, syringe attached to gavage needle, clindamycin, syringe attached to sharp needle).
7	Animal Handler	Administrate dose (antibiotic mix, infection and vancomycin by gavage, clindamycin by intraperitoneal injection).
8	Clean Handler	Close the cage and move it back to the rack
9	Animal Handler	Spread bleach on feces collection tube and change gloves

**Table 3:** Detailed activities to execute in each step

Procedure	Day	Activities
Antibiotic mix (oral gavage)	−6 to −4	Prepare antibiotic mix Collect clinical score and weight Dose by oral gavage
Recovery	−3 to −2	Collect clinical score and weight
Clindamycin (IP)	−1	Prepare clindamycin Collect clinical score and weight Admin clindamycin
Spore challenge	0	Prepare infection inoculum Collect clinical score and weight Admin infection Collect feces
CDI monitoring	1 to 2	Collect clinical score and weight Collect feces
Vancomycin treatment (remission induction)	3 to 7	Prepare vancomycin Collect clinical score and weight Admin vancomycin Collect feces (testing pre-colonization)
R-CDI monitoring	8 to 15	Collect clinical score and weight Collect feces

**Table 4:** Clinical scores

Score	0	1	2	3
Weight loss	Maintained or gain	< 10%	< 15%	< 20%
Appearance	Normal	Lack of grooming	Coat staring, ocular and nasal disc	Piloerection, hunched up
Natural Behavior	Normal	Hyperactivity	Less mobile and alert	Vocalization, restless or still
Provoked Behavior	Normal	Minor depression or exaggerated response	Moderate change in expected behavior	Reacts violently, or very weak
Diarrhea	Normal	Softened stool and color shift	Mucus presence in stool	Liquid stool

Euthanasia end point
Summatory score of 5 or more
Get a score of 3 in any variable
Experience a weight loss of 20%

### Quantification of *C. difficile* colonization in mice feces

**Timing: 2–5 h and 48 h for plate incubation.**

**Figure 2:**
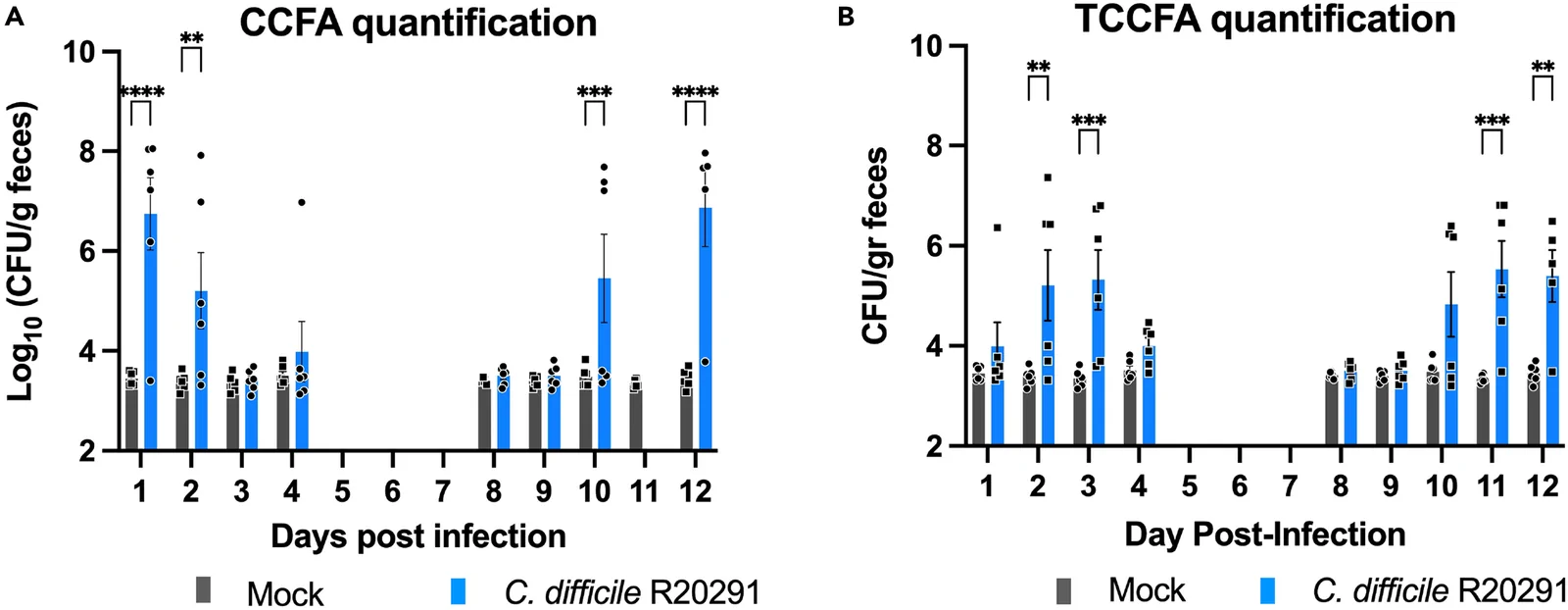
*C. difficile* colonization, quantified in stool suspensions (A) Vegetative cell colonization (homogenized feces and serial dilutions plated in BHIS plates) and (B) spore load (incubation of serial dilutions in ethanol 50% and plated in 0.1% sodium taurocholate-BHIS plates), expressed as log of CFU/g of feces. Multiple comparison, Šídák test, ∗∗ = *p* < 0.005, ∗∗∗ = *p* < 0.001, ∗∗∗∗ = *p* < 0.0001. Error bar represents SEM.

This step describes daily plating of stool suspension to quantify *C. difficile* colonization and spore load, which can be performed daily or at desired timepoints (Figure 2; Table S3).

***Note:*** We recommend collecting feces and quantifying colonization every day of the experiment as a first approach to establish the dynamics of colonization.

9. Record mass of empty pre-weighted tube and mass of tube with stool to calculate stool mass.
10. Place tubes in an anaerobic chamber and add 500 μL of pre-reduced sterile PBS. Incubate for 30 min and shake vigorously to break up the feces.
11. Quantification of vegetative cells (Figure 3):
  a. Spread 100 μL of each sample onto the lower half of a pre-reduced CCFA plate.
  b. Transfer 100 μL of each sample to a 96-well plate.
  c. Dilute the sample 1:10 for four serial dilutions.
  d. Plate 3 μL of each dilution in triplicate on CCFA, including the direct sample.

**Figure 3:**
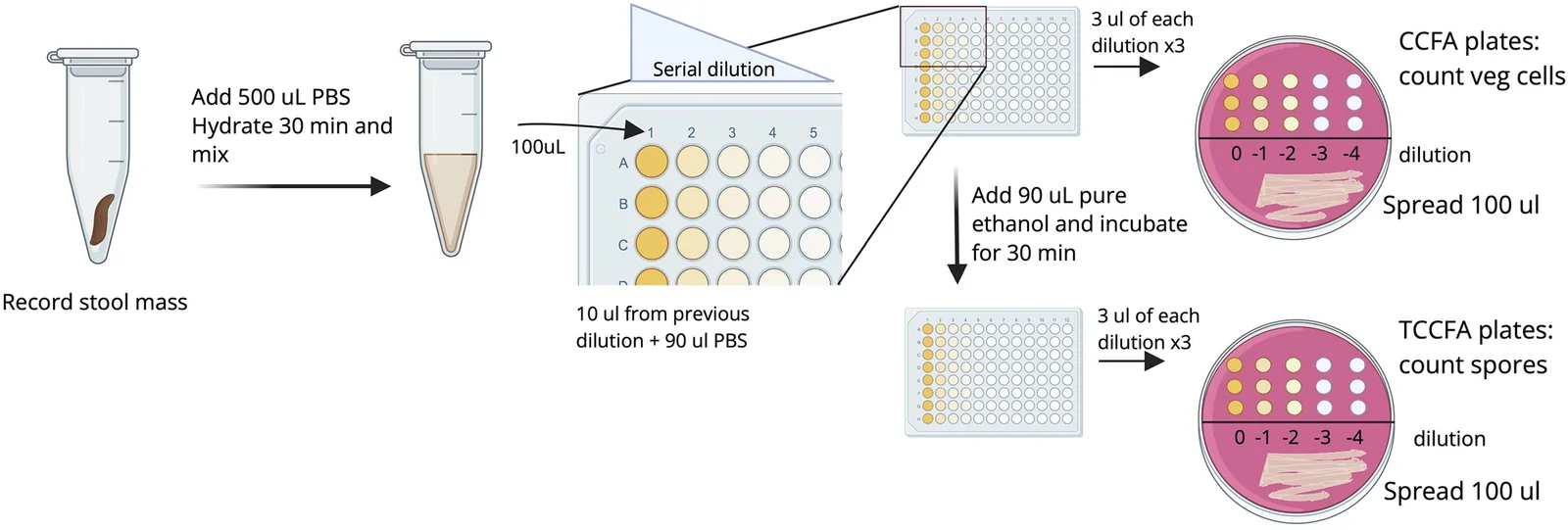
Workflow for quantification of *C. difficile* colonization by stool plating Created in BioRender. Pizarro-Guajardo, M. (2026) https://BioRender.com/y1swdka.
12. Quantification of spores (Figure 3):
  a. Add 90 μL ethanol to each used well of the 96-well plate at a 1:1 ratio of ethanol and incubate for 30 minutes.
  b. Spread 100μL of the undiluted, ethanol-treated sample on TCCFA (Figure 4).

**Figure 4:**
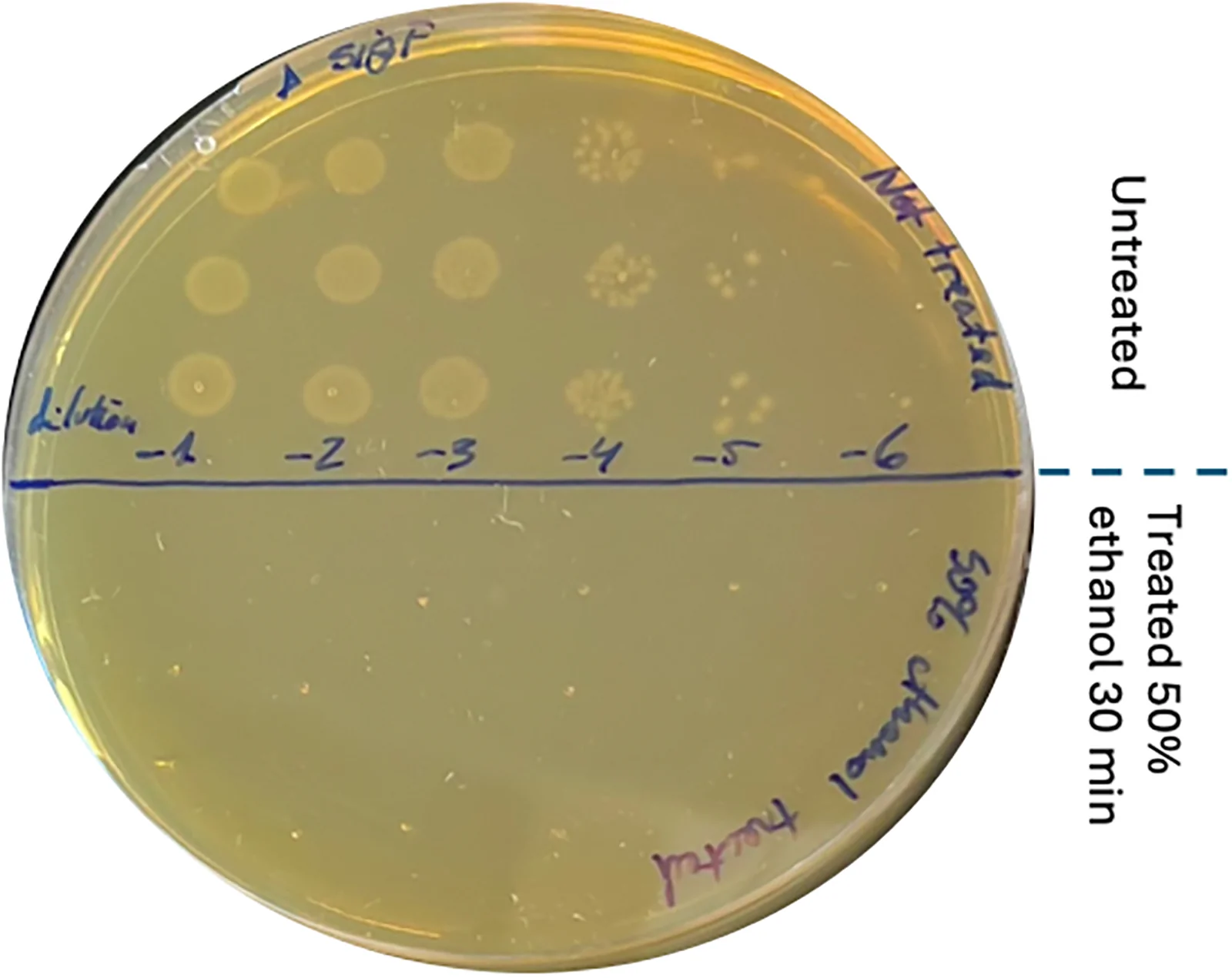
Inactivation of vegetative cells of *C. difficile* with 50% ethanol treatment for 30 min Not treated stationary culture of *C. difficile* mutant in *sigF*, unable to produce spores, serially diluted in PBS and plated on BHIS plates giving 1.67 × 10^8^ CFU/mL. Treatment with ethanol 50% (1:1 mix with 100% ethanol in a well of 96-well plate and incubated for 30 min) followed by plating on 0.1% sodium taurocholate-BHIS agar plate.
  c. Plate 3 μL of each dilution on TCCFA.
13. Incubate all CCFA and TCCFA plates for 48 hours at 37°C in anaerobic chamber.
14. Count and record CFU the day plates are removed from anaerobic chamber. *C. difficile* colonies will clear when exposed to oxygen due to cell lysis.
15. Calculate CFU/g of feces. For CCFA, *CFU/g = CFU/mL × volume of PBS added to initial tube x 1000/mg feces.* For TCCFA, add a ×2 factor to account for the 1:2 dilution with ethanol.

### Ribotyping of *C. difficile* colonies grown on CCFA plates

**Timing: 2 days**

This step describes the characterization of colonizing strains to identify pre-colonization or cross-contamination by ribotyping.

16. Inoculate colonies grown in CCFA into BHIS broth and incubate 16 h at 37°C in an anaerobic chamber.
17. Extract genomic DNA using an appropriate kit or protocol.
18. Run ribotyping PCR.
  a. Prepare master mix for a suitable DNA polymerase, such as Taq polymerase, and add the primers: Forward 16S rRNA “GTGCGGCTGGATCACCTCCT”, Reverse 23S rRNA “CCCTGCACCCTTAATAACTTGACC”.^6^
  b. Run PCR according to DNA polymerase used. For Taq polymerase (NEB, M0267), thermal cycler program is: 1 cycle of 5 min at 95°C for denaturation, followed by 35 cycles (1 min at 95°C, 1 min at 53°C and 1 min at 68°C and final extension of 7 min at 68°C.
  c. Visualize amplification in a TAE – 5% non-denaturing polyacrylamide gels for 45 min at 150 V.

**Alternatives**: This step can be replaced by Fluorescent PCR Ribotyping.^7^

***Note:*** When mutant strains are used, a PCR confirming the genotype can monitor cross-contamination.

**Alternatives**: The *cdeM* gene, encoding a cysteine-rich exosporium protein, is specific to the *C. difficile* lineage^8^ and can be used to identify selected colony cultures as *C. difficile*.

## Expected outcomes

With this protocol we observe development of symptoms in 100% of the animals during primary CDI and 90% during R-CDI. Body weight, clinical scores, and colonization dynamics become consistent after exclusion of pre-contaminated mice by the built-in microbiological checkpoints. Primary *C. difficile* challenge is expected to induce a reproducible, transient weight loss that peaks during acute CDI and partially recovers during vancomycin treatment, with most animals developing diarrhea within 1–2 days after infection and approximately 3 days after cessation of vancomycin in R-CDI (Figure 3). Following vancomycin withdrawal, a defined subset of mice will show renewed weight loss and diarrhea consistent with R-CDI, whereas mock-treated animals maintain stable weight and no clinical signs.

Culture of fecal samples collected at the designated quality-control checkpoints should reveal robust *C. difficile* growth during primary infection and during R-CDI, with minimal or no growth detected in nonrecurrent animals or in pre-screened negative controls, validating the depletion of pre-colonized mice and the effectiveness of cross-contamination control throughout the protocol. In our experience, most of the experiments show no detectable colonization or spore shedding the day after the first dose of vancomycin (Day 4), and plating on these days can be skipped. Fecal cultures should reveal a robust increase in *C. difficile* vegetative burden during acute CDI, with counts typically reaching several log CFU/g and partially declining during remission (Figure 4A). Ethanol-resistant spores are expected to be detectable throughout, with loads that decline more slowly than vegetative cell shedding, providing a quantitative measure of spore carriage in this R-CDI model (Figure 4B).

**Figure 5:**
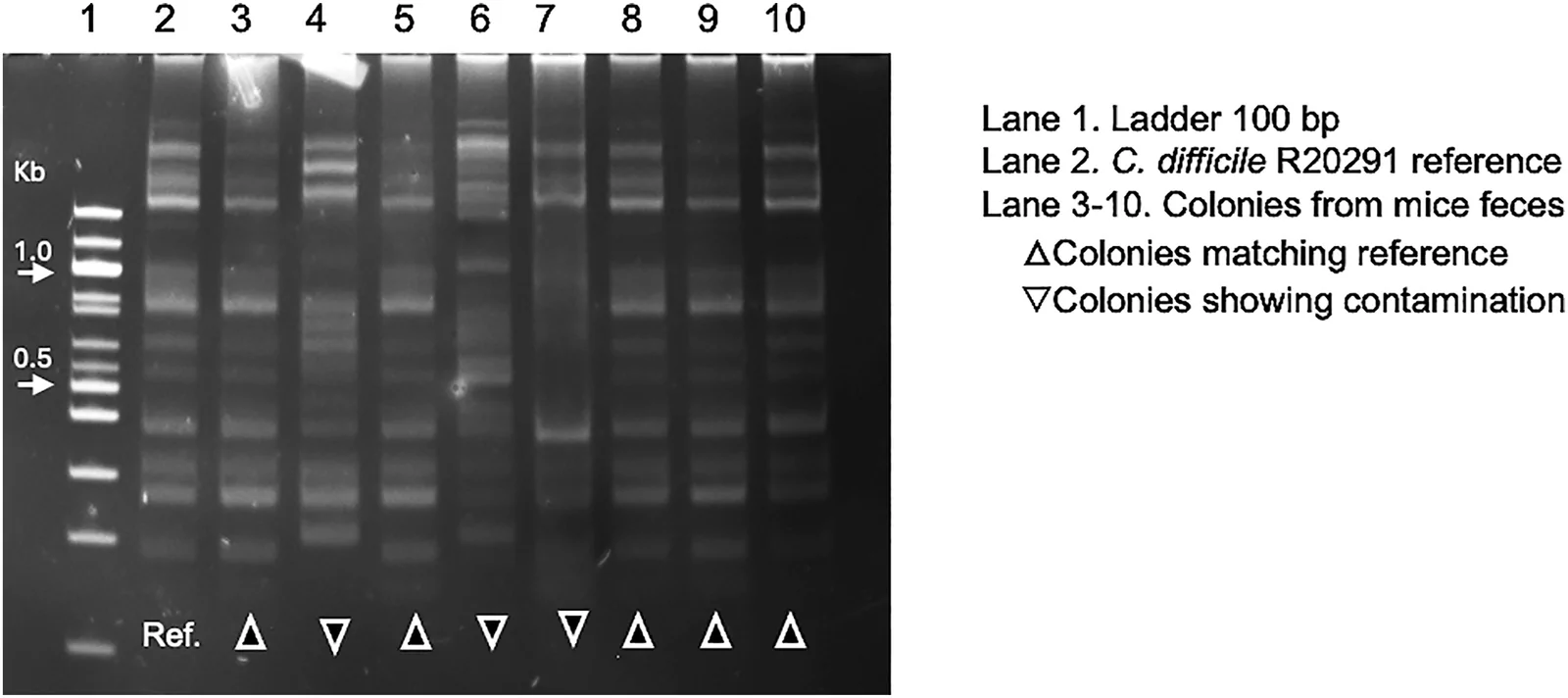
Representative ribotyping PCR Lane 1, 100 bp ladder; lane 2, C. difficile R20291 pattern; lane 3–10, colonies obtained from mice feces. Δ PCR pattern matching reference, ∇ PCR pattern distinctive from reference, showing contamination. Contrast and bright have been adjusted to improve bands clarity.

Ribotyping PCR products run on a polyacrylamide gel allow enough resolution to separate a 100 bp ladder and the amplicons from ribotyping PCR (between 200 and 1.3 kb) should show that most colonies from experimental mice share an identical banding pattern with the *C. difficile* R20291 reference (Figure 5, lanes 3, 5, 8–10, Δ), indicating colonization by the infecting strain. Occasional colonies may display clearly different ribotype profiles (Figure 5, lanes 4,7, ∇), revealing pre-colonization or cross-contamination events that should be documented and excluded from downstream analyses. Similar discriminatory patterns are expected when using fluorescent PCR ribotyping as an alternative readout.

## Limitations

Clinical variables such as weight loss, diarrhea onset, and R-CDI are influenced mostly by mouse strain, vendor and initial microbiota composition; therefore, timing of peak of symptoms and intensity of symptoms can vary at day 2 or 3 after infection. This limitation can be overcome by evaluation of the infective dose. More variability in timing is expected for R-CDI onset, where we have detected that spores stored for longer than 1 month generate delayed CDI due to loss in viability.

Quantification of vegetative cells and spores in the feces provides a relative measure of colonization but may underestimate total *C. difficile*, as vegetative cells are oxygen sensitive and longer times in an oxygenated atmosphere can affect viability. Because a fixed PBS volume is used regardless of fecal mass, the CFU/g detection limit varies between samples: smaller pellets are effectively more diluted, resulting in a higher detection threshold and reduced sensitivity for low-level colonization.

Conventional gel-based ribotyping provides strain-level discrimination but has limited resolution for closely related ribotypes and may miss mixed colonization when minor strains are present at low abundance. The method also requires good-quality PCR and gel separation to resolve banding patterns, and interpretation can be subjective without appropriate reference controls.

## Troubleshooting

### Problem 1

Inconsistent weight loss and clinical scores among a cohort. This can be observed as a low percentage of infected animals experiencing diarrhea or weight loss. Possible causes can be attributed to incomplete antibiotic treatment or variability in mouse intestinal microbiota (Steps 5 and 6).

### Potential solution

Correct dosage: Verify dose calculations and administration volume. Prepare antibiotics fresh every day and reduce storage time when possible. Confirm that intraperitoneal injection is correctly done; we have observed that incorrect administration in intraperitoneal injection considerably reduces the total dose.

Check microbiome dysbiosis: If mouse intestinal microbiota is not sufficiently susceptible to antibiotic treatment before infection, evaluate alternative antibiotic regimens. It has been described that standard diet formulations in combination with the host microbiota can drive variability in severity of infection.^9^ Testing different diets can provide better consistency. In our experience, irradiated food does not require further sterilization if the bag is managed under good practices (use of gloves avoiding touch of dirty surfaces and in sterile environment as under biosafety cabinet).

Correct infective dose: Test several infective doses can be assayed aiming to define a dose that in a particular mice population produces 100% of infection at day 2 or day 3 (peak of infection).

### Problem 2

Inconsistent weight loss and clinical scores between different experiments. This can be observed as variability in the proportion of mice sufficiently infected mice between biological replicates, with different peaks in severity or onset of infection. Possible cause includes lack of consistency in the infective dose between batches and animal experiments, or aging of the spore stock.

### Potential solution

Evaluate the viability of the infective dose, expressed as CFU/mL, before infection to standardize the stock of infective spores. Re-evaluate viability of the infective dose a second time after infection, to quantify the diluted spore suspension used and ensure consistency among biological replicates.

### Problem 3

Low feces collection on days with severe symptoms, resulting in low or zero CFU quantification.

### Potential solution

If less than 10 mg of feces is collected, hydrate with 200 μL of PBS and adjust the CFU/g formula accordingly. By reducing the volume, the limit of detection is lowered, ensuring a sufficient concentration to quantify colonies.

### Problem 4

Difficulty obtaining feces. Mice are unable to provide fresh fecal pellets at the time of collection.

### Potential solution

Manipulate animals and collect feces at the same time every day to reduce variability and improve handling efficiency. In our experience, 1 hour after the light is on, there is an optimal 3 hours window during which feces can be collected.

### Problem 5

Different colony morphologies on CCFA and TCCFA plates. These cefoxitin and cycloserine resistant colonies have distinct morphological differences to *C. difficile* colonies.

### Potential solution

**Figure 6:**
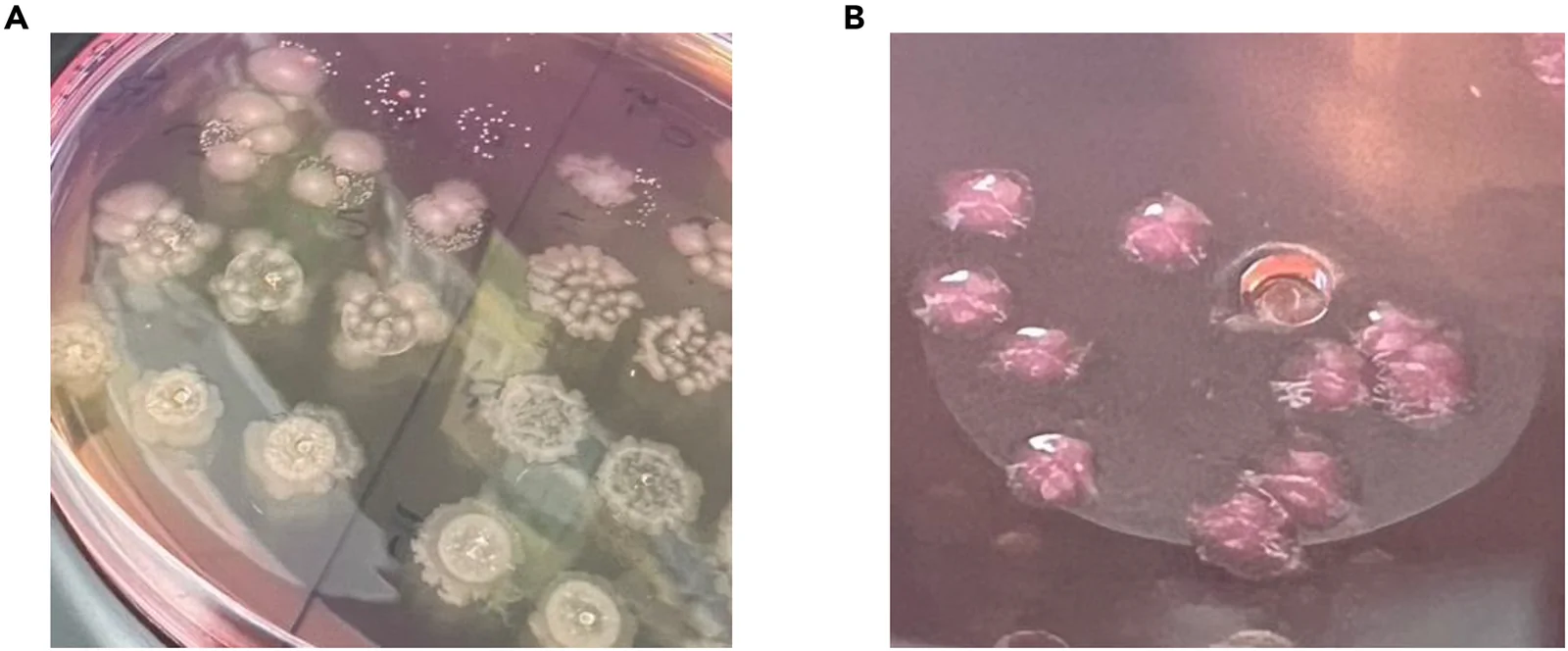
Distinctive colony morphology of *C. difficile* R20291 and comparison with other colonies found in CCFA and TCCFA (A) *C. difficile* colonies appear slightly raised in the center, transparent, with irregular, filamentous borders and a characteristic yellow discoloration of the surrounding agar after growth, distinguishing them from (B) bright, smooth-edged contaminant colonies.

Store CCFA and TCCFA at 4°C and ensure that they are fresh (optimally < 2 week old). Visual analysis of colonies should match the infecting strain colony morphology (different strains and ribotypes can have different colony morphologies). To ensure accurate counting, extract gDNA from colony derived stationary phase cultures and perform a ribotype and *cdeM* PCR to confirm the presence of the infecting strain (Figure 6).

## Resource availability

### Lead contact

Further information and requests for resources and reagents should be directed to and will be fulfilled by the lead contact, Daniel Paredes-Sabja (dparedes-sabja@tamu.edu).

### Technical contact

Technical questions on executing this protocol should be directed to and will be answered by the technical contact, Marjorie Pizarro-Guajardo (mpizarro-guajardo@tamu.edu).

### Materials availability

This study did not generate new material.

### Data and code availability

This study did not generate new code. Data are included as supplemental material.

## Acknowledgments

This work was supported by 10.13039/100000002NIH founding (10.13039/100000060NIAID, 5R01AI177842) to D.P.-S. and the Dementia & Alzheimer’s Research Initiative (DARI) founds to M.P.-G. The graphical abstract and Figure 3 were created with BioRender.com.

## Author contributions

M.P.-G. optimized animal handling protocols, optimized plating and ribotyping protocols, collected and managed data, drafted the article, and edited the final manuscript. T.H. optimized animal handling protocols, optimized the infection, collected and analyzed data, and reviewed the article. D.P.-S. reviewed the protocols, the study design, and the article.

## Declaration of interests

The authors declare no competing interests.
