# Supplementary material for: Protocol to study recurrence of Clostridioides difficile infection in murine model with built-in colonization and contamination controls
Source: STAR Protoc. 2026 Aug 22;7(3):104746. doi: 10.1016/j.xpro.2026.104746 (PMC13524870; doi:10.1016/j.xpro.2026.104746)
Supplement: Supplementary file 1 [file mmc1.pdf]

Table S1. Data weight loss, related to Figure 1B.

	Mock						*C. difficile* R20291					
Day	Mouse 1	Mouse 2	Mouse 3	Mouse 4	Mouse 5	Mouse 6	Mouse 1	Mouse 2	Mouse 3	Mouse 4	Mouse 5	Mouse 6
0	100	100	100	100	100	100	100	100	100	100	100	100
1	103.061224	102.199345	101.170117	99.8268898	101.950427	102.547393	101.342009	101.27193	99.8809996	97.6494291	96.875	98.9314896
2	102.96846	102.480112	101.980198	102.36584	104.794799	103.317536	93.3712891	89.7807018	91.4716382	95.7018133	100.3125	102.451288
3	99.3506494	100.280767	98.919892	98.2688979	100.934579	102.547393	90.4839366	88.8157895	92.4236414	96.8435191	99.75	102.576996
4	98.0055659	100.327562	98.6948695	97.6341604	100.893946	100.35545	96.6246442	91.5350877	94.7243157	94.35863	100.1875	98.2401006
5	98.8404453	99.9064109	97.9747975	96.7686093	100.650142	96.6232227	98.4139894	94.5175439	95.8746529	96.373405	99.0625	97.485858
6	100.742115	103.041647	100.675068	100.980958	101.584722	97.8672986	101.098007	97.1052632	97.3819913	95.4331766	101.1875	100.502828
7	100.556586	101.731399	99.5049505	98.9036353	100.487607	98.0450237	98.4139894	96.4912281	95.9936533	97.7837475	97.6875	101.194217
8	102.179963	102.714085	101.125113	103.577611	104.225924	101.184834	99.6339976	99.2105263	96.7473225	99.7313633	98.8125	103.268385
9	104.267161	104.117922	103.060306	106.693595	104.063389	101.836493	101.260675	99.7368421	98.6909956	99.7985225	102.5625	103.079824
10	103.710575	103.837155	104.725473	106.174264	102.803738	101.954976	102.196015	99.122807	99.3653312	101.61182	102.75	104.211188
11	96.2894249	98.9705194	103.510351	104.904789	103.372613	104.265403	96.2586417	89.122807	93.7723126	93.8885158	104.5	95.7259585
12	97.3562152	94.1974731	103.870387	103.981535	102.92564	104.561611	92.4359496	83.377193	Died	100.470114	106.375	100.628536
13	104.267161	94.6654188	105.130513	109.001731	101.381552	108.412322	96.5026434	87.5438596		104.566823	105.3125	101.94846
14	104.823748	98.7833411	104.815482	110.386613	105.404307	108.056872	97.1533144	91.5789474		102.484889	105.9375	105.782527
15	104.823748	102.573701	105.310531	112.002308	103.941487	109.537915	99.6746645	95.3508772		101.880457	106.875	107.919547
16	104.545455	102.199345	103.19532	111.425274	105.810646	110.42654	100.488003	97.1929825		105.842848	108.0625	111.313639

Table S2. Data time to diarrhea in CDI and R-CDI, related to Figure 1C.

Time to diarrhea, CDI. Days 1 to 4

Day	Mock	*C. difficile* R20291
3	1	
3	1	
4	0	
4	0	
4	0	
4	0	
1		1
1		1
1		1
2		1
2		1
3		0

Time to diarrhea, CDI. Days 1 to 4

Day	Mock	*C. difficile* R20291
11	1	
12	1	
14	1	
14	0	
14	0	
14	0	
11		1
11		1
11		1
11		1
11		1
13		1

Table S3. Log$_{10}$ of CFU/g of feces, obtained in CCFA and TCCFA, related to Figure 2.

Log$_{10}$ (CFU/g feces) from CCFA												
Day	Mock						*C. difficile* R20291					
	Mouse 1	Mouse 2	Mouse 3	Mouse 4	Mouse 5	Mouse 6	Mouse 1	Mouse 2	Mouse 3	Mouse 4	Mouse 5	Mouse 6
1	3.32	3.59	3.61	3.31	3.35	3.54	6.19	3.40	8.03	8.05	7.59	7.23
2	3.43	3.29	3.46	3.64	3.14	3.39	3.31	4.55	6.98	7.92	3.52	4.96
3	3.31	3.37	3.21	3.62	3.45	3.14	3.40	3.27	3.10	3.44	3.59	3.69
4	3.31	3.82	3.46	3.69	3.43	3.37	3.49	3.20	3.14	6.98	3.64	3.46
8	3.38	3.49	3.42	3.39	3.33	3.48	3.33	3.25	3.64	3.57	3.69	3.54
9	3.25	3.49	3.37	3.51	3.43	3.32	3.81	3.22	3.39	3.59	3.65	3.36
10	3.32	3.33	3.32	3.54	3.56	3.83	7.21	3.36	3.50	7.38	3.59	7.68
11	3.24	3.46	3.42	3.31	3.32	3.30	7.24	7.84	4.29	7.50	3.65	7.69
12	3.18	3.39	3.35	3.52	3.57	3.70	7.24	7.97	*	7.66	3.78	7.70
Log$_{10}$ (CFU/g feces) from TCCFA												
Day	Mock						*C. difficile* R20291					
1	3.32	3.59	3.61	3.31	3.35	3.54	3.32	3.40	3.59	6.36	3.77	3.50
2	3.43	3.29	3.46	3.64	3.14	3.39	3.31	4.01	6.44	7.36	6.43	3.70
3	3.31	3.37	3.21	3.62	3.45	3.14	4.96	6.74	6.80	6.14	3.59	3.69
4	3.31	3.82	3.46	3.69	3.43	3.37	3.89	4.29	4.24	4.47	3.64	3.46
8	3.38	3.49	3.42	3.39	3.33	3.48	3.33	3.25	3.64	3.57	3.69	3.54
9	3.25	3.49	3.37	3.51	3.43	3.32	3.81	3.22	3.39	3.59	3.65	3.36
10	3.32	3.33	3.32	3.54	3.56	3.83	6.39	3.36	3.20	6.24	3.59	6.18
11	3.24	3.46	3.42	3.31	3.32	3.30	5.13	6.81	4.50	6.81	3.48	6.46
12	3.18	3.39	3.35	3.52	3.57	3.70	5.27	6.11	*	6.49	3.48	5.64
